# Transcriptomic and metabonomic profiling unveils the mechanism of Tartary buckwheat and kiwi co-fermentation products in hyperlipidemia treatment

**DOI:** 10.3389/fphar.2025.1572593

**Published:** 2025-05-30

**Authors:** Nan Guohui, Xie Tingna, He Qinghui, Yu Hongchun, Peng Jing, Jiang Shiyin, Li Li, Yuan Huawei, Wei Daijing, Wu Qi

**Affiliations:** ^1^ Faculty of Quality Management and Inspection and Quarantine, Yibin University, Yibin, Sichuan, China; ^2^ Yibin Center of Inspection and Testing, Yibin, China; ^3^ College of Life Science, Sichuan Agricultural University, Sichuan, China

**Keywords:** Tartary buckwheat -kiwi fruit, flavonoids, transcriptomics and metabolomics, anti-hyperlipidemic effects, zebrafish, fermentation

## Abstract

**Introduction:**

Tartary buckwheat (*Fagopyrum tataricum, TB*), a flavonoid-rich plant limited by anti-nutritional metabolites.

**Methods:**

TB was co-fermented with kiwi juice (TB-KW) through alcohol fermentation to improve flavonoid extraction and utilization. The flavonoid profile of TB-KW was analyzed through untargeted metabolomics. The anti-hyperlipidemic effects of TB-KW were assessed in zebrafish maintained on a high-fat diet through transcriptomics and metabolomics.

**Results:**

Untargeted metabolomic analysis showed that flavonoids originating from TB, including quercetin, luteolin, quercitrin, rutin, and kaempferide, were significantly enriched in TB-KW. The data further showed that TB-KW significantly reduced lipid accumulation in zebrafish. Metabolomic profiling revealed 24 core differential metabolites (DEMs), spanning glycerophospholipids, sphingolipids, glycerolipids, and fatty acyls. Transcriptome analysis showed that TB-KW significantly regulated genes such as *PLTP, ApoC1, SOAT2, SCARB1, PLA2G12B,* and *HMGCRa*.

**Discussion:**

These genes are associated with cholesterol metabolism and pathways linked to fat digestion and absorption, and they show a particular capacity to increase HDL synthesis. This study suggests the potential of TB-KW in improving flavonoid bioavailability and in the prevention and treatment of hyperlipidemia.

## Introduction

Hyperlipidemia is a metabolic disorder characterized by abnormal lipid metabolism, marked by elevated levels of total cholesterol (TC), triacylglycerol (TG), and low-density lipoprotein cholesterol (LDL-C) in the blood or decreased levels of high-density lipoprotein cholesterol (HDL-C) (Min et al., 2024). This condition is associated with an increased risk of cardiovascular diseases (CVDs), including atherosclerosis, coronary heart disease, and stroke (Wu et al., 2024). Common LDL-C-lowering medications, such as niacin, resins, and statins, often lead to adverse reactions, primarily headaches, respiratory infections, musculoskeletal pain, and gastrointestinal issues (Zhang et al., 2022). Health foods rich in secondary metabolites have attracted significant interest due to their potential role in supporting lipid metabolism.

Flavonoids are a prominent group of secondary metabolites in plants, known for their anti-hyperlipidemic, anti-diabetic, antioxidant, and anti-inflammatory properties (Lukas, 2023). For instance, flavonoid extracts from *Citrus aurantium* have been shown to regulate the expression levels of *AMPK-α1*, *PPARα*, and *PPARγ* in mice fed a high-fat diet (Lin et al., 2024). Multi-omics analyses have demonstrated that genes, metabolites, and gut microbiota associated with lipid metabolism can be influenced by baicalein in hyperlipidemic mice (Ping et al., 2022). Beyond flavonoids, the lipid-lowering effects of polyphenols and vitamin C (Vc) have been well-documented in various studies (Lin et al., 2022b; Li et al., 2022). Consequently, the development of foods enriched with functional components has become a focal point in the adjuvant treatment of hyperlipidemia.

*Fagopyrum tataricum* (L.) Gaertn. (Tartary buckwheat, TB) is a unique pseudocereal, rich in starch, that serves both medicinal and culinary purposes (Cheng et al., 2023; Wang et al., 2023; Gavrić et al., 2023). TB is reported to have protective effects on the cardiovascular system, largely due to its high flavonoid content (Wang et al., 2024). Consequently, TB-based products such as noodles, tea, alcoholic beverages, and vinegar are gaining popularity among consumers. The hypolipidemic effects of TB-derived pasta and oil have been demonstrated in rodents fed a high-fat diet (Xin et al., 2023; Nicolò et al., 2014). TB products are typically made from its seeds, however, their development faces limitations because TB contains anti-nutritional factors the seeds are difficult to shell (Ying et al., 2022). Research indicates that TB shells contain even higher concentrations of flavonoids (Yuan and Duan, 2017). Additionally, while flavonoids generally exhibit limited solubility in water, they dissolve more readily in alcohol.

To address these limitations, ethanol fermentation of whole TB offers an effective processing approach capable of resolving the challenges mentioned above. In this study, TB was co-fermented with vitamin C-rich *Actinidia chinensis* Planch (kiwi) to provide additional sugars, improve the sensory characteristics, and increase functional properties during fermentation. The objective was to assess the utilization of flavonoids in the resulting product (TB-KW), evaluate its effects on abnormal lipid metabolism, and investigate the related mechanisms. This work contributes to the development of ethanol-fermented TB products with improved nutritional and functional value.

## Materials and methods

### Preparation of TB-KW

*Fagopyrum tataricum* (L.) Gaertn. was sourced from Xichang, Sichuan Province, China. *Actinidia chinensis* Planch was obtained from Yibin, Sichuan Province, China. The tartary buckwheat to kiwi mass ratio was maintained at 1:9. Optimal fermentation conditions for TB-KW were established as follows: yeast (AngelYeast Co., Ltd., China) inoculation at 1.5 g/L, initial sugar content 21%, initial pH 4.2, fermentation temperature 25°C, and a fermentation duration of 7 d. After fermentation, the TB-KW reached an alcohol concentration of 12% vol. For this study, three bottles were randomly selected from separate batches, and all experiments were carried out in triplicate to maintain consistency.

### Determination of nutritional and functional metabolites of TB-KW

To preliminarily assess the quality of TB-KW, we analyzed the main nutritional and functional metabolites along with antioxidant activity. The contents of reducing sugar, total acids, alcohol, soluble solids, and Vitamin C (Vc) were determined following methods detailed in previous reports (Qiang et al., 2023; Al-Sultani and Ajeena, 2023; Yanbo Liu et al., 2022). Protein content was measured colorimetrically using the Coomassie Brilliant Blue method (Sedmak and Grossberg, 1977), while total flavonoid content was determined colorimetrically using the aluminum nitrate method, with kiwi serving as a control (Qinyuan et al., 2020). The antioxidant capacity of TB-KW was evaluated by measuring its anti-DPPH radical activity and by obtaining redox potentials, estimated via the FRAP method (Čakar et al., 2019).

### Metabolites analysis of TB-KW using untargeted techniques

To investigate the influence of TB on the TB-KW’s composition, untargeted analytical methods were employed to identify metabolite differences between kiwi juice after fermentation (KW) and TB-KW. Specifically, 100 μL of each sample was transferred to a 2 mL centrifuge tube and vortex-mixed for 60 s. Samples were then centrifuged at 12,000 rpm for 10 min at 4°C. After centrifugation, the supernatant was filtered through a 0.22 μm PTFE microporous membrane and prepared for liquid chromatography-mass spectrometry (LC-MS) analysis.

The LC analysis was performed on a Vanquish UHPLC System (Thermo Fisher Scientific, United States). Chromatography was carried out with an ACQUITY UPLC ^®^ HSS T3 (2.1 × 100 mm, 1.8 µm) (Waters, Milford, MA, United States). The column maintained at 40 °C. The flow rate and injection volume were set at 0.3 mL/min and 2 μL, respectively. For LC-ESI (+)-MS analysis, the mobile phases consisted of (B2) 0.1% formic acid in acetonitrile (v/v) and (A2) 0.1% formic acid in water (v/v). Separation was conducted under the following gradient: 0–1 min, 8% B2; 1–8 min, 8%–98% B2; 8–10 min, 98% B2; 10–10.1 min, 98%–8% B2; 10.1–12 min, 8% B2. For LC-ESI (−)-MS analysis, the analytes was carried out with (B3) acetonitrile and (A3) ammonium formate (5 mM). Separation was conducted under the following gradient: 0–1 min,8% B3; 1–8 min,8%–98% B3; 8–10 min, 98% B3; 10–10.1 min, 98%–8% B3; 10.1–12 min, 8% B3.

Mass spectrometric detection of metabolites was performed on Orbitrap Exploris 120 (Thermo Fisher Scientific, United States) with ESI ion source. Simultaneous MS1 and MS/MS (Full MS-ddMS2 mode, data-dependent MS/MS) acquisition was used. The parameters were as follows: sheath gas pressure, 40 arb; aux gas flow, 10 arb; spray voltage, 3.50 kV and −2.50 kV for ESI (+) and ESI (−), respectively; capillary temperature, 325°C; MS1 range, m/z 100–1,000; MS1 resolving power, 60,000 FWHM; number of data dependant scans per cycle, 4; MS/MS resolving power, 15,000 FWHM; normalized collision energy, 30%; dynamic exclusion time, automatic.

Quality control (QC) samples were created by pooling all individual samples to form a composite sample. Each experiment was conducted in triplicate to ensure reproducibility and reliability of the results. Metabolite data were collected and analyzed according to established protocols (Li et al., 2024b). Metabolites identified in TB-KW were input into the Swiss ADME database (http://www.swissadme.ch/) for further evaluation. Active metabolites were screened based on criteria of “GI absorption = High” and “Druglikeness ≥ 2 YES”. Potential targets of TB-KW were identified using the SwissTargetPrediction platform (http://www.swisstargetprediction.ch) (Li et al., 2024a).

### Zebrafish maintenance

Albino adult zebrafish (*Danio rerio*) were obtained from Hangzhou Hunter Biotechnology Co., LTD., (Hangzhou, China) with ethical approval from the Ethical Committee for Animal Experiments at Yibin University. The fish were kept at 28°C in brine under a 12-h light/12-h dark cycle. They were fed standard commercial fish feed twice daily at a quantity equal to 1% of their body weight, and the water was refreshed every 3 days to ensure optimal conditions.

### Determination of maximum tolerant concentration

To establish the maximum tolerated concentration (MTC) of TB-KW in zebrafish and determine the appropriate therapeutic dosage, albino zebrafish larvae at 5 days post-fertilization (dpf) were placed into five beakers. The larvae were fed a high-fat diet (HFD) composed of egg yolk powder (Zhejiang Aige Biotechnology Co., LTD., China) at a 4% ratio with normal fish feed (NFD), along with TB-KW at various final concentrations (1.25, 2.50, 5.00, 10.0, and 20.0 μL/mL). Zebrafish on a high-fat diet alone served as the model group, while those on a normal diet were designated as the control group. After 48 h of exposure, the MTC was determined based on mortality rates.

### Feeding and treatment method of zebrafish

Atorvastatin (ATV), a widely used statin for managing hyperlipidemia, was obtained in 10 mg tablet form from Pfizer (New York, NY, United States). Albino zebrafish larvae at 5 dpf were randomly divided into six groups: NFD, HFD, HFD2.5, HFD5, HFD10, and HFD-ATV. The NFD group received a normal diet, while the HFD group was given a high-fat diet. The HFD2.5, HFD5, and HFD10 groups were fed the same high-fat diet and treated with TB-KW at final concentrations of 2.5 μL/mL, 5 μL/mL, and 10 μL/mL, respectively, added to the fish water. The HFD-ATV group received the high-fat diet with ATV added to the fish water at a final concentration of 11.6 μL/mL. Each group consisted of 30 fish, with no distinction made between sexes.

### Triglyceride content measurement

After 48 h of treatment at 28°C, the triglyceride (TG) content in zebrafish was determined. Whole-body fat was stained using Oil Red O, and 10 zebrafish from each group were randomly selected for imaging under a dissecting microscope (SZX7, Olympus, Japan). The intensity of staining in the tail vessels was quantified using NIS-Elements D 3.20 software.

### Cholesterol content measurement

Following 32 h of treatment at 28°C, total cholesterol (TC) levels were measured. Zebrafish from the NFD, HFD, and HFD10 groups were injected with cholesterol probes (Invitrogen, United States) and then incubated for an additional 16 h. Ten zebrafish were randomly selected from each group, and cholesterol fluorescence intensity in the tail vessels was analyzed using a fluorescence microscope (AZ100, Nikon, Japan) and NIS-Elements D 3.20 software.

### Determination of reactive oxygen species

Reactive oxygen species (ROS) levels were assessed after 2 days of treatment at 28°C. Ten zebrafish from the NFD, HFD, and HFD10 groups were placed in a 6-well plate (Bioland, China) and washed three times with water. Each well was filled to a volume of 2 mL with ROS fluorescence detection solution. Zebrafish were then transferred to a 96-well plate, and ROS fluorescence intensity was measured using a multifunctional microplate reader (SPARK, TECAN, Switzerland).

### Transcriptomic analysis

Groups NFD, HFD, and HFD10 were selected for transcriptomic analysis. After 48 h of treatment, 30 zebrafish from each group were collected, flash-frozen in liquid nitrogen for 3 min, and stored at −80°C. Three biological replicates were established in parallel. Total RNA was extracted, and each sample’s concentration, purity, and integrity were assessed. Transcriptome sequencing library preparation, clustering, and sequencing were carried out by Hangzhou Hunter Biotechnology Co., Ltd. Genes with an absolute log2 fold change ≥ 1 and a Q-value ≤ 0.05 were defined as differentially expressed genes (DEGs) and included in downstream analysis. Raw sequencing data were further processed using a bioinformatics pipeline on the BGI online platform (https://biosys.bgi.com).

### Lipidomic analysis

Groups NFD, HFD, and HFD10 were also subjected to lipidomic analysis. Following 48 h of treatment at 28°C, 30 zebrafish per group were collected, rapidly frozen in liquid nitrogen for 3 min, and stored at −80°C. Three biological replicates were prepared in parallel. For each replicate, 20 mg of zebrafish tissue was extracted using 1.0 mL of MTBE/methanol (3:1, v/v). Samples were homogenized using a mixer mill (Retsch, Haan, Germany) with zirconia beads, then vortexed for 2 min and sonicated for 5 min. To separate the phases, 200 μL of water was added, followed by vortexing for 1 min and centrifugation at 12,000 rpm for 10 min at 4°C. From the upper organic phase, 300 μL was carefully collected, evaporated to dryness, and reconstituted in acetonitrile/isopropanol (1:9, v/v) for LC-ESI-MS/MS analysis.

Lipidomic profiling and preliminary data processing were performed by Metware Biotechnology (Wuhan, China). Mass spectrometric data were analyzed using Analyst 1.6.3 software (AB Sciex). For metabolite identification, both the proprietary Metware database (MWDB) and public databases were consulted. Structural annotation of lipid metabolites was based primarily on MassBank, KNAPSAcK, HMDB, Lipidmaps, and METLIN. Metabolites with VIP scores ≥1.0 and fold changes ≥2 or ≤ 0.5 were classified as differentially abundant metabolites (DAMs). Further analyses were conducted using KEGG, MetaboAnalyst, and R software.

### Integration analysis of the metabolomic and transcriptome

Network analysis using bioinformatics was performed through the Cytoscape plugin, MetaboAnalyst (Junli et al., 2023). IDs of differentially expressed genes (DEGs) and metabolites obtained from metabolomic and transcriptomic profiling were imported into MetScape (Jagadish et al., 2010). A metabolite-Gene network type was selected to construct the network, which generated associated networks and a list of relevant pathways by querying applicable databases.

### Determination of gene expression levels

To validate the transcriptomic analysis results, total mRNA was extracted from zebrafish in the NFD, HFD, and HFD10 groups (n = 6) following the RNAout kit instructions (Tianze, China). First-strand cDNA synthesis was performed using the RevertAid™ First Strand cDNA Synthesis Kit (MBI, United States) with oligo (dT) primers, following the kit’s methodology. PCR primers used are listed in Supplementary Table S1.

### Statistical analysis

Data were presented as mean ± standard deviation (SD). Differences among multiple groups were evaluated using a one-way analysis of variance (ANOVA), followed by Tukey’s *post hoc* test for further comparison.

## Results

### The content of nutritional and functional metabolites in TB-KW

The nutritional and functional metabolites of TB-KW used in this study were analyzed. The content of reducing sugar, total acids, protein, total flavonoid content, and Vitamin C (Vc) were showen in Table 1. The total flavonoid content in TB-KW was significantly higher (*p* < 0.05) than that in KW (0.02 ± 0.00 mg/mL). The total flavonoid and Vc contribute to the antioxidant properties of TB-KW. The anti-DPPH radical activity was measured at 37.7%, and the total antioxidant capacity reached 88.6%, both significantly higher than those in KW (*p* < 0.05). These findings suggest that TB-KW has promising functional potential, warranting further validation through animal experiments.

**TABLE 1:** The content of nutritional and functional components in TB-KW.

Components	Content
Reducing sugar	6.35 ± 0.03 mg/mL
Total acids	13.48 ± 0.27 g/L
Protein	29.81 ± 0.12 μg/mL
Total flavonoid	0.15 ± 0.01 mg/mL
Vitamin c	78.43 ± 0.16 mg/100 mL

### Tartary buckwheat changed the metabolites of TB-KW

**FIGURE 1:**
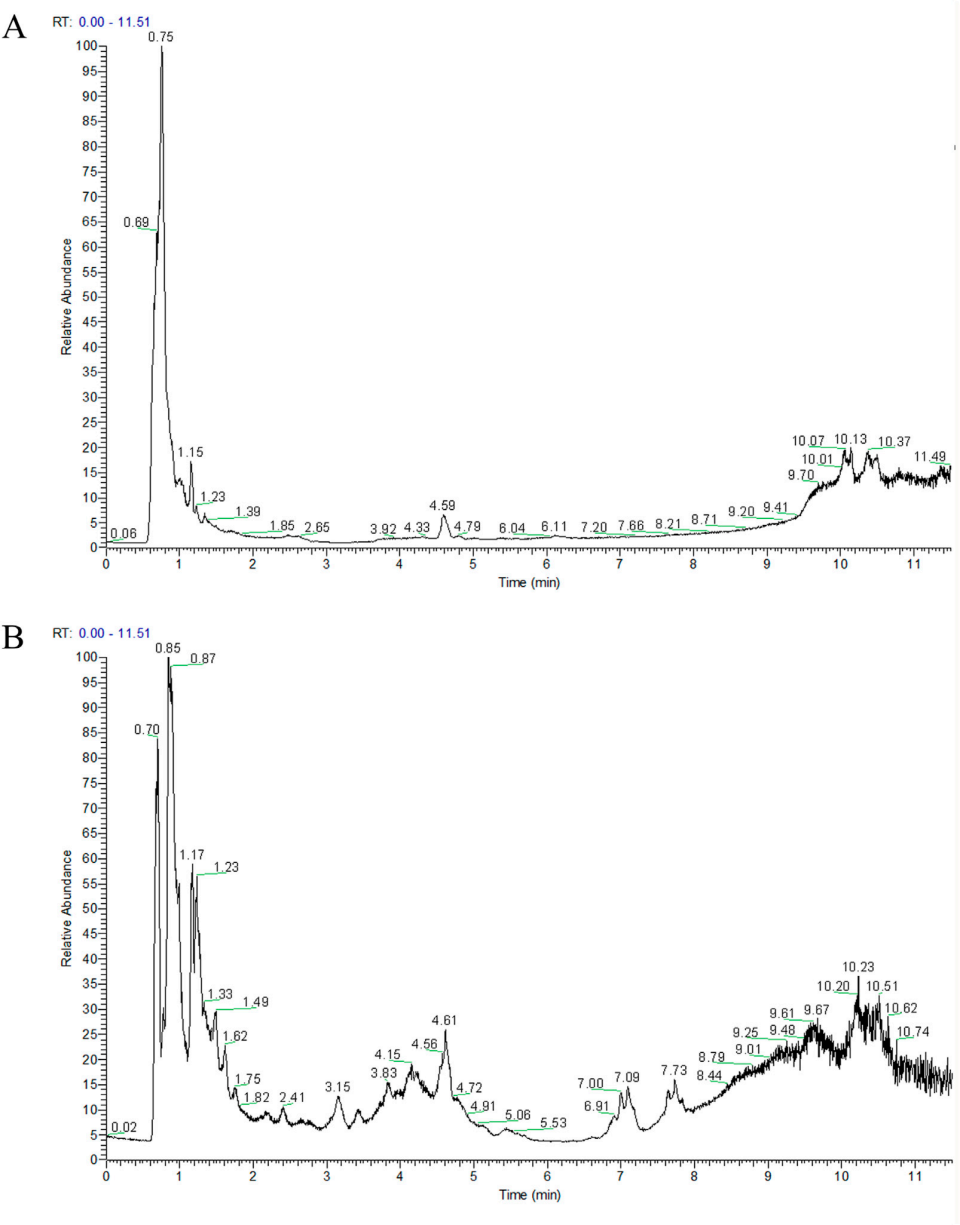
TIC diagram of negative ion **(A)** and positive ion **(B)** modes of compounds from TB-KW decoction.

Overall, 335 metabolites of TB-KW were identified using PANOMIX Biomedical Tech’s proprietary database and their unique secondary mass spectrometry fragmentation pattern matching technique. Of these, 114 were detected in the negative ion mode (Figure 1A) and 221 in the positive ion mode (Figure 1B). These metabolites were categorized into various groups, including flavonoids (11), prenol lipids (8), organooxygen metabolites (32), carboxylic acids and derivatives (50), fatty acyls (33), benzene and substituted derivatives (19), and 182 other metabolites.

**FIGURE 2:**
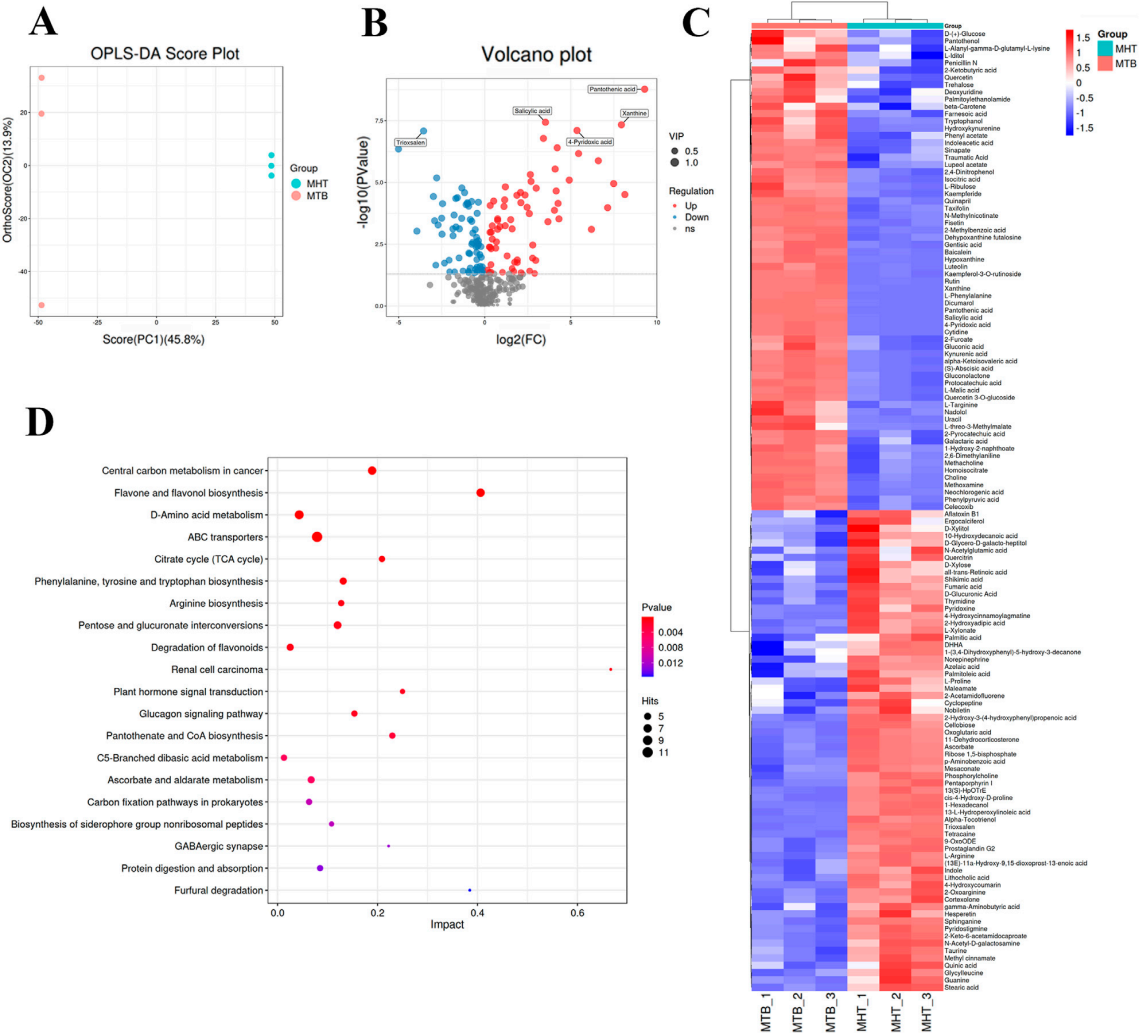
The effects of TB on metabolites in TB-KW. **(A)** OPLS-DA scores plots of MTB versus MHT. **(B)** Volcano plot analysis of DEMs in MTB versus MHT. **(C)** heat map of the DEMs. **(D)** KEGG pathway enrichment analysis of DEMs in the MTB group that have significant differences with MHT group. The lipid species with significant differences were identified by a criterion which was FC ≥2 or ≤0.5 and VIP ≥1 in the volcano plot. The red (upregulated) dots and blue (downregulated) dots represent the DEMs (n = 3). The y-axis indicates the KEGG pathway names. The dot size means the gene number. The dot color indicates the q value. The top 20 KEGG pathways are presented.

The effects of Tartary buckwheat on the metabolites in TB-KW were assessed using untargeted techniques. An OPLS-DA model was first applied to reduce the dimensionality of metabolite data and improve interpretability (Figure 2A). Results indicated clear differences between sample groups, with PC1 and PC2 jointly accounting for 59.7% of the variation between groups. Further univariate statistical analysis, including t-tests (*p* < 0.05) and fold-change analysis (FC ≥2 or ≤0.5), was conducted between the MTB and MHT groups. Differential metabolites (DEMs), including unidentified metabolites, were highlighted in a Volcano Plot and heat map (Figures 2B,C). A total of 132 DEMs were identified, with 66 upregulated and 66 downregulated (Supplementary Figure S1; Supplementary File 1). KEGG pathway enrichment analysis of the DEMs revealed that, compared with kiwi fermentation alone, co-fermentation notably enhanced pathways such as central carbon metabolism in cancer, flavone and flavonol biosynthesis, D-amino acid metabolism, and flavonoid degradation (Figure 2D; Supplementary File 2). Notably, metabolites enriched in the flavonoid metabolic pathway included quercetin, luteolin, quercitrin, quercetin 3-O-glucoside, rutin, kaempferide, and kaempferol-3-O-rutinoside. These findings suggest that Tartary buckwheat contributes positively to the fermentation of TB-KW by supplying carbon sources, amino acids, and flavonoids. However, further studies are necessary to assess the safety and lipid-lowering efficacy of TB-KW.

### MTC of TB-KW

As shown in Table 2, zebrafish treated with TB-KW displayed no observable phenotypic abnormalities at concentrations from 1.25 to 10 μL/mL. However, at a concentration of 20 μL/mL, 67% mortality was observed (20 out of 30 zebrafish). Based on these findings, subsequent experiments were conducted using TB-KW concentrations of 2.5, 5, and 10 μL/mL.

**TABLE 2:** Evaluation results of safe concentration of TB-KW.

Group	Concentration (μL/mL)	Death number(N)	Mortality (%)	Phenotypes
NFD	—	0	0	No observable abnormality
HFD	—	0	0	No observable abnormality
HFD and TB-KW	1.25	0	0	Similar to the HFD group
	2.50	0	0	Similar to the HFD group
	5.00	0	0	Similar to the HFD group
	10.0	0	0	Similar to the HFD group
	20.0	20	67	—

### TB-KW improved lipid metabolism disorders induced by high-fat diet

**FIGURE 3:**
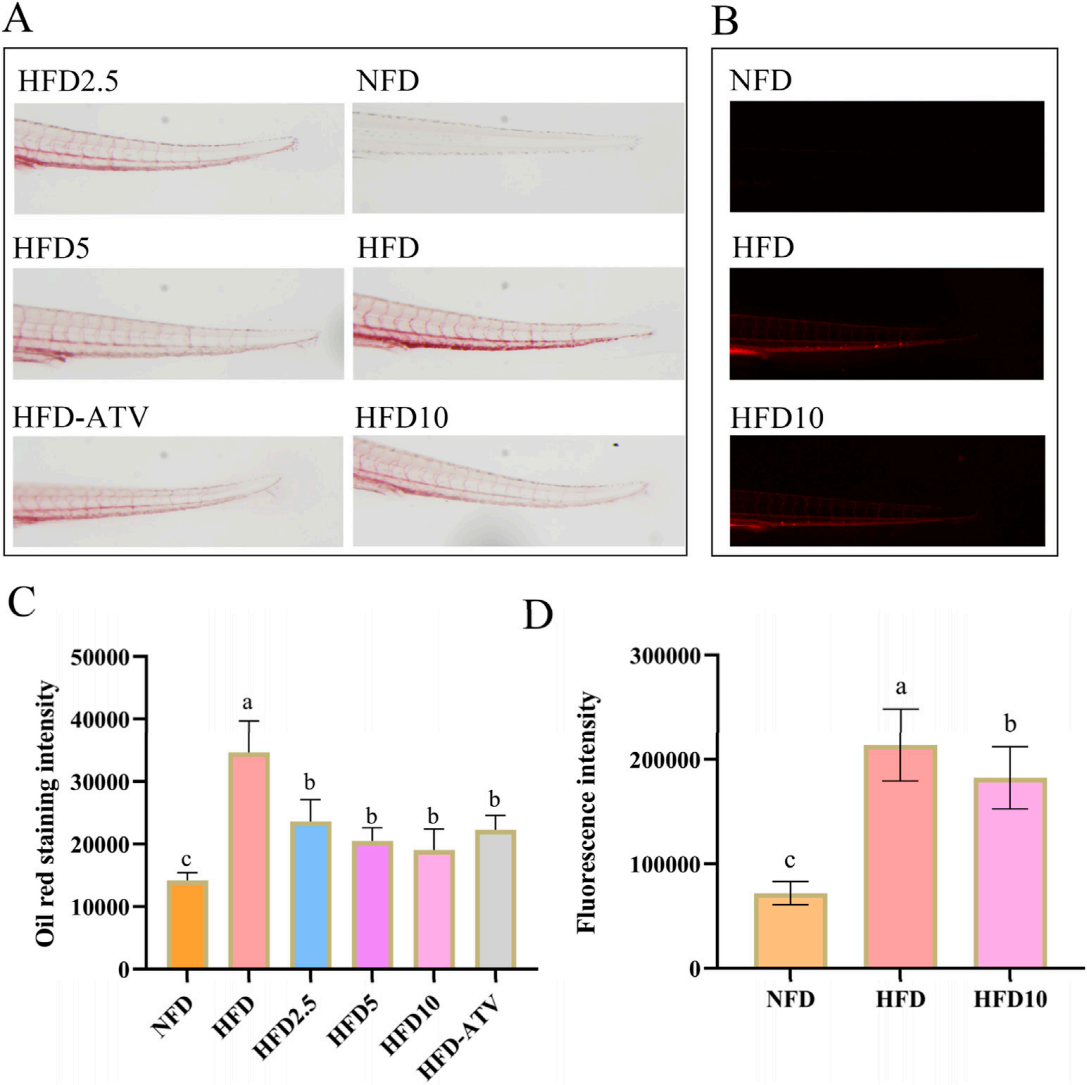
TB-KW treatment ameliorated high-fat diet-induced abnormal lipid metabolism in zebrafish. **(A)** Oil Red O-stained images of the blood vessels. **(B)** TC fluorescence staining of the blood vessels. **(C)** Oil Red O staining intensity. **(D)** Fluorescence intensity. Data are shown as means ± SD (n = 10). Different letters on the data indicate significant differences compared with the other groups (*p* < 0.05).

To evaluate the effect of TB-KW on lipid metabolism, Oil Red O staining and cholesterol probe assays were employed to measure triglyceride (TG) and total cholesterol (TC) levels in zebrafish fed a high-fat diet (Figure 3). TC and TG levels were significantly higher in the high-fat diet (HFD) group than in the normal-fat diet (NFD) group (*p* < 0.05). Treatment with TB-KW led to a significant reduction in both TC (Figures 3A, 2C) and TG (Figures 3B,D) levels in the HFD group (*p* < 0.05). The reduction in TC was comparable to that achieved with ATV. As shown in Figure 3C, TC levels declined with increasing doses of TB-KW, indicating a dose-dependent effect. These results suggest that TB-KW effectively improves lipid profiles disrupted by high-fat feeding.

### TB-KW improves the antioxidant capacity of the body

**FIGURE 4:**
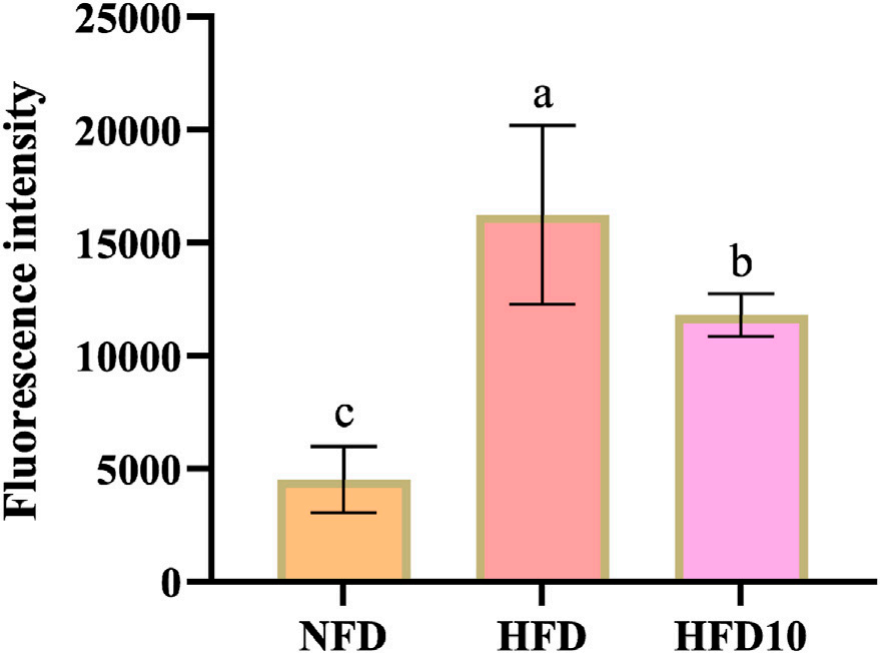
ROS fluorescence intensity of zebrafish.

Tartary buckwheat and kiwifruit are known for their antioxidant properties. Hence, this study evaluated the effectiveness of TB-KW in reducing ROS levels. Compared to the NFD group, the high-fat diet (HFD) group exhibited a significant increase in ROS levels (*p* < 0.05). However, the intake of TB-KW effectively countered this rise, reversing the trend (Figure 4). These findings suggest that TB-KW has a strong capacity to combat oxidative damage.

### TB-KW reverse HFD-induced transcriptome alterations

**FIGURE 5:**
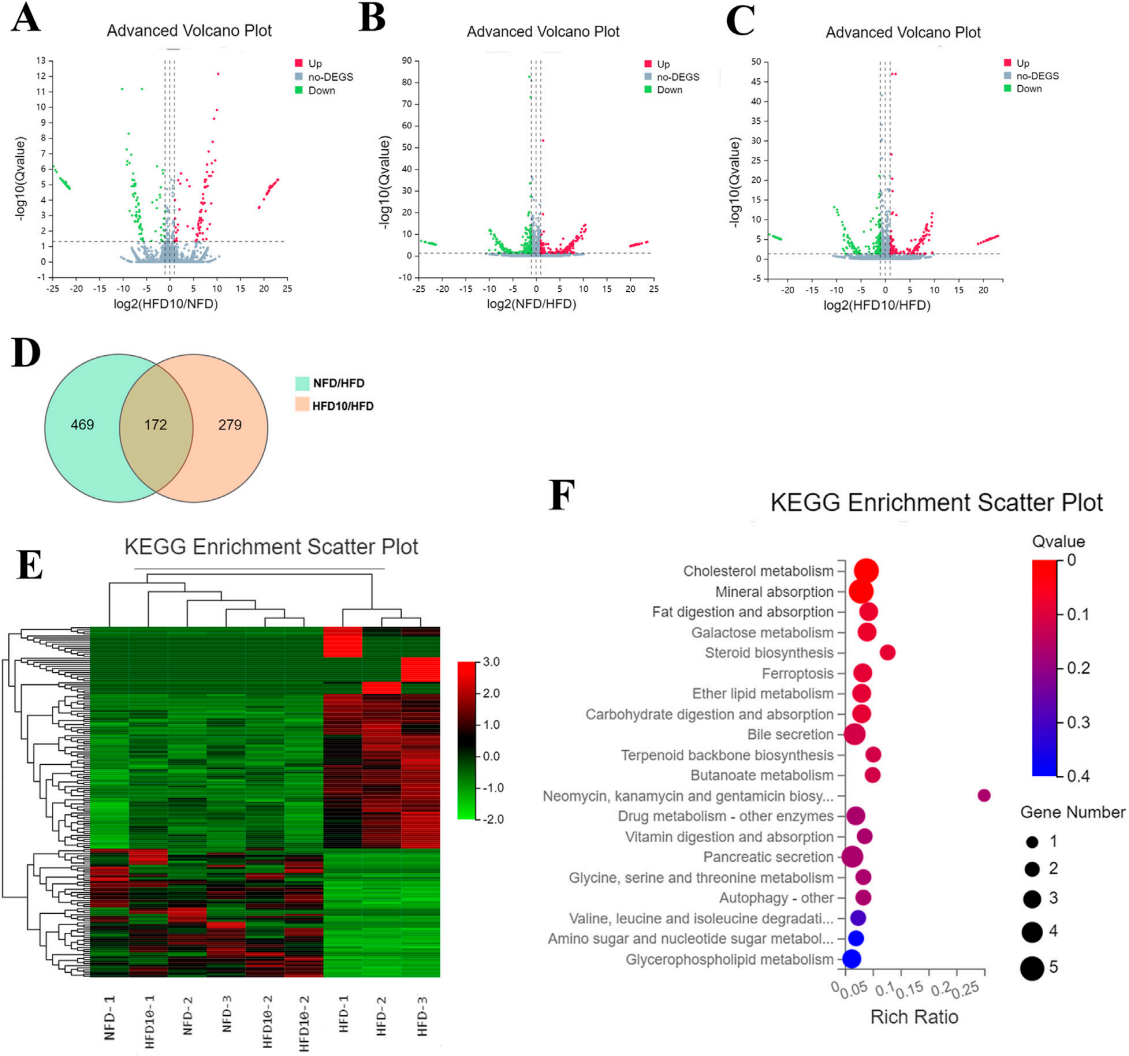
Transcriptome analysis of zebrafish samples. Volcano plot analysis of significantly differential genes in HFD10 versus NFD **(A)**, NFD versus HFD **(B)**, and HFD10 versus HFD **(C)**. DEGs of genes among the three different groups were shown in Venn diagrams **(D)** and the heat map **(E)**. KEGG pathway enrichment analysis **(F)** of DEGs in the HFD10 group that have significant differences with HFD while less differences with NFD group. Significantly DEGs were selected by using the criteria of FC ≥1 and *p* < 0.05 in the volcano plot (n = 3). Significantly differential genes were shown as a red (up) or green (down) dot, a gray dot represented no significant differences. The y-axis indicates the KEGG pathway names. The dot size means the gene number. The dot color indicates the *q* value. The top 20 KEGG pathways are presented.

To explore gene expression changes in hyperlipidemic zebrafish induced by a high-fat diet (HFD) and treated with TB-KW, RNA-Seq analysis was conducted. A total of 69,201 transcripts met the quality requirements. Using a threshold of fold change ≥1 and *p* ≤ 0.05, 641 differentially expressed genes (DEGs) were identified between the HFD and NFD groups, including 261 upregulated and 380 downregulated genes. Comparison of the HFD10 group (treated with 10 μL/mL TB-KW) to the HFD group revealed 451 DEGs, with 191 genes upregulated and 260 downregulated. Between the HFD10 and NFD groups, 257 DEGs were found, with 121 upregulated and 136 downregulated. These results are visualized in the volcano plots in Figures 5A–C.

The heatmap of DEGs showed clear differences in gene expression across the three groups. The transcriptomic profile of the HFD10 group was more similar to that of the NFD group than to the HFD group (Figure 5E). As shown in the Venn diagrams (Figure 5D), TB-KW restored the expression of 172 specific genes in the HFD10 group when compared to the HFD group (Supplementary File 3). Of the 109 genes upregulated by the high-fat diet, TB-KW treatment reduced their expression to below the levels seen in the HFD group. Additionally, TB-KW reversed the downregulation of 63 genes caused by the HFD, bringing their expression closer to that of the NFD group.

KEGG pathway analysis was then used to identify the metabolic pathways associated with the 172 DEGs. The 20 pathways with the lowest q-values are shown in Figure 5F. When comparing the NFD and HFD10 groups to the HFD group, key enriched pathways in carbohydrate and lipid metabolism included cholesterol metabolism, fat digestion and absorption, galactose metabolism, steroid biosynthesis, ether lipid metabolism, carbohydrate digestion and absorption, terpenoid backbone biosynthesis, butanoate metabolism, and glycerophospholipid metabolism. In addition, pathways such as bile secretion, pancreatic secretion, and autophagy were also among the top 20, pointing to broader metabolic effects of the fruit. The corresponding genes within these pathways are detailed in Supplementary File 4. Notably, genes such as *PLTP*, *ApoC1*, *SOAT2*, *SCARB1*, *PLA2G12B*, and *HMGCRa* were found to play key roles in cholesterol metabolism and fat digestion and absorption. To confirm the RNA-Seq findings, qRT-PCR was performed on selected genes. The expression trends of four genes (Supplementary Figure S2) were consistent with the RNA-Seq results.

### TB-KW intake improves lipid metabolomic profiling

**FIGURE 6:**
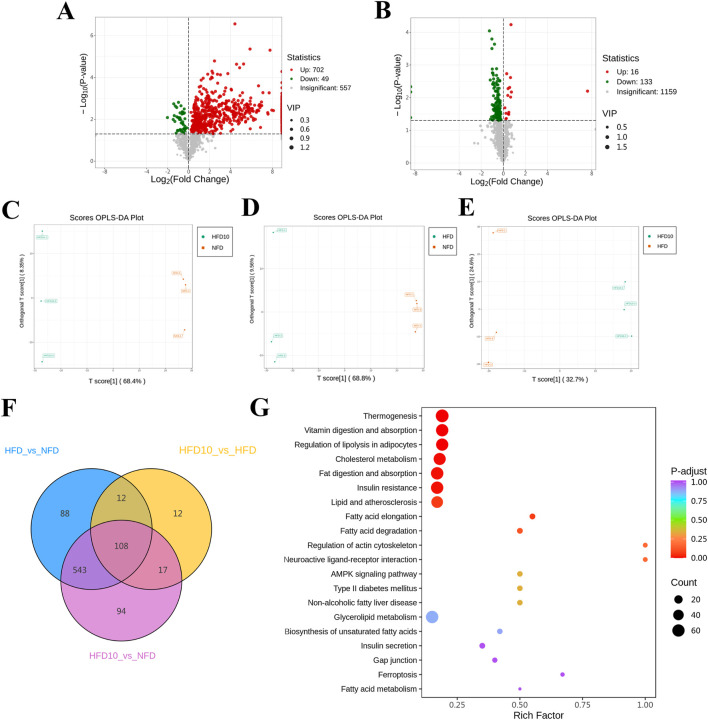
Lipidomic analysis of zebrafish samples. **(A,B)** Volcano plot analysis of significantly differential genes in HFD versus NFD, HFD10 versus HFD. **(C–E)** OPLS-DA scores plots of HFD10 versus HFD, HFD versus NFD, and HFD10 versus HFD. **(F)** Venn diagrams analysis. **(G)** Lipid metabolic pathway analysis based on the 149 screened lipid species. The lipid species with significant differences were identified by a criterion which was FC ≥ 2 or ≤0.5 and VIP ≥ 1 in the volcano plot. The red (upregulated) dots and green (downregulated) dots represent the lipid species with significant difference (n = 3).

To examine the relationship between lipid biomarkers and the biological activity of TB-KW, lipidomic analysis was performed to assess lipid composition differences in zebrafish with and without TB-KW intake. Two multivariate OPLS-DA models were constructed to compare the groups and evaluate variability. Model validation showed strong fit and predictability metrics: for the HFD versus NFD comparison, R2X = 0.784, R2Y = 1.000, Q2 = 0.968; and for the HFD10 versus HFD comparison, R2X = 0.573, R2Y = 0.998, Q2 = 0.712. These values indicate that OPLS-DA provided a dependable model for distinguishing between lipidomic profiles. Both sets of comparisons displayed clear group separations in the OPLS-DA score plots (Figures 6A,B).

Out of 1,154 significant lipid species, 776 in the HFD10 group shifted in a direction consistent with the NFD group, suggesting that TB-KW intake substantially counteracted lipid metabolic disruptions caused by the high-fat diet. DEMs were identified as potential lipid biomarkers through volcano plots (Figures 6C–E) and Venn diagram analysis (Figure 6F). Using a threshold of fold change (FC ≥2 or ≤0.5) and variable importance in projection (VIP ≥ 1), 851 lipid species showed significant differences between the HFD and NFD groups. Supplementary Figure S3 presents the top 50 lipids with the highest VIP values. Among the 149 lipids showing differences between the HFD10 and HFD groups, 24 were significantly altered in HFD10 compared to HFD but not compared to NFD (Supplementary File 5), indicating that these 24 lipids may act as markers of TB-KW’s lipid-lowering potential.

To identify the related biochemical and signaling pathways, enrichment analysis was conducted on the 149 significantly altered lipid species. As shown in Figure 6G, TB-KW reduced pathway disruptions induced by the high-fat diet, influencing thermogenesis, vitamin digestion and absorption, regulation of lipolysis in adipocytes, cholesterol metabolism, fat digestion and absorption, insulin resistance, lipid metabolism and atherosclerosis, fatty acid elongation, and fatty acid degradation.

### Integrating transcriptomics and metabolomics data

**FIGURE 7:**
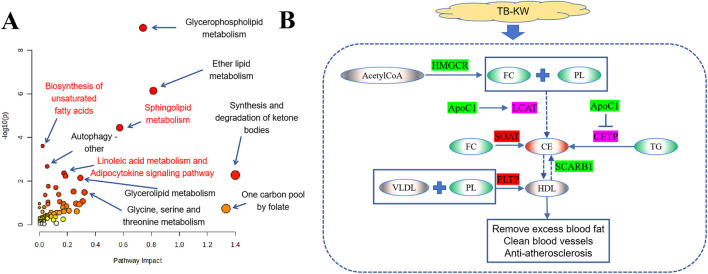
Integrating transcriptomics and metabolomics data. **(A)** Bubble diagram of the significant KEGG pathway for DEGs and DAMs identified between the experimental groups. Color of the dots represents the level of significance, with yellow being the least and red being the most significant. Size of the dots represents the pathway impact values from the pathway topology analysis, and the range of the impact values is 0.00–1.00. **(B)** Possible mechanisms of TB-KW supplementation preventing from abnormal lipids metabolism in zebrafish induced by HFD. Ellipse denotes compound, square denotes gene; red indicates upregulation, green indicates downregulation, purple indicates upregulation but no significant difference, and gray indicates no data support. FC, Cholesterol; PL, Phospholipid; CE, Cholesterol ester.

To further understand the metabolic pathways affected by TB-KW supplementation, an integrative analysis of transcriptomic and metabolomic data was conducted. The pathway map, manually compiled, is presented in Figure 7A. Results indicate that the network of 172 DEGs and 149 DAMs is primarily enriched in pathways such as glycerophospholipid metabolism, ether lipid metabolism, sphingolipid metabolism, biosynthesis of unsaturated fatty acids, linoleic acid metabolism, autophagy, synthesis and degradation of ketone bodies, adipocytokine signaling, glycerolipid metabolism, fatty acid elongation, PPAR signaling, glycosylphosphatidylinositol-anchor biosynthesis, and glycine, serine, and threonine metabolism (Supplementary File 6). Based on predicted target analysis, 30 active metabolites in TB-KW were identified as potential regulators of key genes, including *SOAT2*, *SCARB1*, *PLA2G12B*, and *HMGCR* (Supplementary Table S2). The consistency observed between transcriptomic and metabolomic data suggests that TB-KW may contribute to elevating HDL levels (Figure 7B), although further experimental validation is required to confirm this finding. Overall, this integrative analysis of transcriptomic and metabolomic data indicates that TB-KW may alleviate high-fat diet-induced dyslipidemia by modulating lipid metabolism, autophagy, and pathways involved in oxidative stress resistance.

## Discussion

Tartary buckwheat is well-known for its high flavonoid content, with metabolomic studies identifying 147 flavonoids and a total flavonoid concentration of 2.5 mg/g (Pan et al., 2022; Siyu et al., 2021). The most abundant flavonoids in Tartary buckwheat include rutin, quercetin, and kaempferol, all detected as DEMs in TB-KW. This presence supports the enhanced antioxidant properties of TB-KW, directly linked to Tartary buckwheat’s inclusion, thereby improving the’s functional potential. Kiwi fruit, rich in vitamin C, folate, vitamin E, dietary fibers, antioxidants, enzymes, and phytonutrients, contains 125 flavonoids with a total flavonoid content of 1.51 mg/g (Yu et al., 2020; Miguel et al., 2021). These qualities make both Tartary buckwheat and kiwi promising candidates for health food development.

Fruit-based alcoholic beverages, rich in polyphenols, flavonoids, and other antioxidants, have been shown to reduce cellular damage associated with inflammation and oxidative stress (Antika et al., 2021). The antioxidant properties of Tartary buckwheat and kiwi have been repeatedly confirmed (Gavrić et al., 2023; Lin et al., 2022a). In this study, we examined the functional metabolites and antioxidant potential of TB-KW and observed that it effectively normalized disrupted lipid metabolism and restored SOD levels, indicating its capacity to counter oxidative damage in the body. Additionally, TB-KW was found to be safe for zebrafish at concentrations below 10 μL/mL, which is roughly equivalent to a daily intake of 100 mL for humans.

Previous studies have highlighted the role of both kiwi and Tartary buckwheat in improving lipid metabolism. Kiwi has been shown to regulate lipid levels in hyperlipidemic mice by downregulating genes associated with inflammation and lipid synthesis while upregulating those linked to energy use (Xiaoni et al., 2021). Likewise, Tartary buckwheat has demonstrated the ability to counter lipid metabolism disorders and inflammation triggered by a high-fat diet through pathways involving AMPK, SREBP, and NF-kappa B (Ping et al., 2023). In our study, we identified several genes—*PLTP*, *ApoC1*, *SOAT2*, *SCARB1*, *PLA2G12B*, and *HMGCRa*—involved in cholesterol metabolism, fat digestion and absorption, and insulin resistance. The lipid-regulating effects observed here are supported by prior findings showing that flavonoids such as rutin and quercetin exhibit strong binding affinities with HMGCR (Guo et al., 2020).

Metabolomic evidence has further demonstrated kiwi’s capacity to regulate hyperlipidemia through pathways related to vitamin digestion and absorption, pyrimidine metabolism, purine metabolism, and others (Kan et al., 2023). Similarly, Tartary buckwheat has been shown to ease high-fat diet-induced metabolic disturbances by influencing 30 metabolites across eight pathways, including linoleic acid and arachidonic acid metabolism (Xiaoli et al., 2022). In our study, we identified 149 lipid species, primarily including TGs (triacylglycerols), PGs (phosphatidylglycerols), PEs (phosphatidylethanolamines), and LPSs (lysophosphatidylserines). These lipids are closely linked to cholesterol metabolism, fat digestion and absorption, and insulin resistance.

Numerous epidemiological studies have shown that moderate alcohol consumption can increase overall life expectancy by reducing the risk of atherosclerotic vascular disease (Goldberg and Soleas, 2011). Our findings indicate that TB-KW has beneficial regulatory effects on lipid metabolism, including cholesterol metabolism, fat digestion, and absorption pathways. Additionally, TB-KW has potential HDL-increasing properties, as also observed in functional evaluations of *Rosa roxburghii* and haihong alcoholic drinks (Hui et al., 2021; AN et al., 2022). However, the mechanisms by which TB-KW influences lipid metabolism may vary. One likely reason for differences between our findings and those of previous studies is that fermentation affects the composition of raw materials. Notably, no metabolic abnormalities related to alcohol consumption were observed in the HFD10 group.

Plant-derived natural metabolites can be absorbed into the animal body through the digestive tract. For instance, quercetin and rutin are absorbed at different sites along the gastrointestinal tract (Kammalla et al., 2015). Increasing evidence suggests that these metabolites interact with key genes involved in lipid metabolism. Studies have shown that both rutin and quercetin accumulate in the liver and exhibit strong binding affinity with HMGCR (Guo Zhuang et al., 2021). This indicates that metabolites derived from TB-KW may directly influence genes related to lipid regulation. However, flavonoids undergo both dosage form transformation and chemical modification in the gastrointestinal tract and liver, leading to the formation of multiple metabolites. These secondary metabolites may also influence genes associated with lipid metabolism (Chen et al., 2022). Therefore, the link between TB-KW intake, shifts in the metabolomic profile, and transcriptomic responses is complex and requires further investigation to clarify the mechanisms involved.

In conclusion, this study demonstrates that TB-KW effectively regulates abnormal lipid metabolism and provides antioxidant benefits. Using metabolomic and transcriptomic analyses, we confirmed that TB-KW regulates the expression of *PLTP*, *ApoC1*, *SOAT2*, *SCARB1*, *PLA2G12B*, and *HMGCRa*, improves levels of TGs, PGs, PEs, and LPSs, and influences cholesterol metabolism and related pathways. These findings highlight the potential of TB-KW to improve flavonoid utilization and reduce lipid levels, providing a theoretical foundation for developing and promoting Tartary buckwheat resources.

## Data Availability

The data presented in the study are deposited in the NCBI repository, accession number PRJNA1263917. Further inquiries can be directed to the corresponding author.

## Supplementary material

SUPPLEMENTARY FILE 1 DEMs identified between MTB and MHT groups.

SUPPLEMENTARY FILE 2 KEGG pathway enrichment analysis of the DEMs between MTB and MHT Groups.

SUPPLEMENTARY FILE 3 DEGs identified between HFD10 and HFD groups.

SUPPLEMENTARY FILE 4 KEGG pathway enrichment analysis of the DEGs between HFD10 and HFD groups.

SUPPLEMENTARY FILE 5 DEMs identified between HFD10 and HFD groups.

SUPPLEMENTARY FILE 6 Integrating transcriptomics and metabolomics data.

## References

[B1] Al-Sultani H. A. F. Ajeena S. J. (2023). Identification of some phenolic compounds of purslane extracts (Portulaca Oleraceae L.) and evaluation its content from vit.E and vit.C and its antioxidant activity in Iraq. IOP Conf. Ser. Earth Environ. Sci. 1262, 062039. 10.1088/1755-1315/1262/6/062039

[B2] Antika B. Sumaporn K. Kraireuk N. Pilanee V. Waraporn A. Chanaporn T. (2021). Fermentation condition and quality evaluation of pineapple fruit wine. Fermentation 8, 11. 10.3390/fermentation8010011

[B3] An Y. Zhou J. Zhu D. (2022). Rosa roxburghii fruit wine improves glucose and lipid metabolism disorder in type 2 diabetic rats. Shipin gongye ke-ji, 361–368. 10.13386/j.issn1002-0306.2021100316

[B4] Čakar U. Petrović A. Pejin B. Čakar M. Živković M. Vajs V. (2019). Fruit as a substrate for a wine: a case study of selected berry and drupe fruit wines. Sci. Hortic. 244, 42–49. 10.1016/j.scienta.2018.09.020

[B5] Cheng T. Wang Q. Ma C. Gan Z. Wan Y. Ye X. (2023). Study on the growth dynamics of tartary buckwheat flowers and grains, as well as material basis and physiological changes of their seed-setting differences. Agronomy 14, 49. 10.3390/agronomy14010049

[B6] Chen L. Cao H. Huang Q. Xiao J. Teng H. (2022). Absorption, metabolism and bioavailability of flavonoids: a review. Crit. Rev. Food Sci. And Nutr. 62, 7730–7742. 10.1080/10408398.2021.1917508 34078189

[B7] Gavrić T. Matijević A. Šakonjić A. Bezdrob M. (2023). The influence of fertilisation on the yield and antioxidant capacity of common and tartary buckwheat. Agric. and For./Poljoprivreda i šumarstv 69, 7–18. 10.17707/AgricultForest.69.4.01

[B8] Goldberg D. Soleas G. (2011). Wine and health: a paradigm for alcohol and antioxidants. J. Med. Biochem. 30, 93–102. 10.2478/v10011-011-0003-9

[B9] Guo Z. Yuqing W. Shujiao L. Xu J. Xiaoyu W. (2020). Tissue distribution and molecular docking research on the active components of Bidens bipinnata L. against hyperlipidemia. Biomed. Chromatogr. 35, e5026. 10.1002/bmc.5026 33169423

[B10] Hui Y. Wen S. Junli H. Chuang W. Chaoyun W. (2021). Investigation of non-volatile substances in the Haihong fruit wine and their lipid-lowering effect. Food Biosci. 39, 100836. 10.1016/j.fbio.2020.100836

[B11] Jagadish H. V. Omenn G. S. Tarcea V. G. Karnovsky A. Beecher C. W. Weymouth T. E. (2010). Metscape: a Cytoscape plug-in for visualizing and interpreting metabolomic data in the context of human metabolic networks. Bioinformatics 26, 971–973. 10.1093/bioinformatics/btq048 20139469 PMC2844990

[B12] Junli F. XI C. Shitong W. Jian Z. Qingcheng W. Shunyuan G. (2023). Transcriptomics integrated with metabolomics reveals the ameliorating effect of mussel-derived plasmalogens on high-fat diet-induced hyperlipidemia in zebrafish. Food and Funct. 14, 3641–3658. 10.1039/d3fo00063j 36961308

[B13] Kammalla A. K. Ramasamy M. K. Chintala J. Dubey G. P. Agrawal A. Kaliappan I. (2015). Comparative pharmacokinetic interactions of Quercetin and Rutin in rats after oral administration of European patented formulation containing Hipphophae rhamnoides and Co-administration of Quercetin and Rutin. Eur. J. Drug Metabolism Pharmacokinet. 40, 277–284. 10.1007/s13318-014-0206-9 24888486

[B14] Kan H. Qian G. Jinxing S. Hai S. Xia M. Shangquan J. (2023). Gut microbiome and metabolomics study of selenium-enriched kiwifruit regulating hyperlipidemia in mice induced by a high-fat diet. J. Agric. food Chem. 71, 20386–20401. 10.1021/acs.jafc.3c00108 38055355

[B15] Lin C. Mei W. Y. Ju S. G. (2022a). Variations of antioxidant properties and enzymes activities during the whole fruit fermentation of two kiwifruit (;Actinidia chinenesis;Planch.). Int. J. Fruit Sci. 22, 860–871. 10.1080/15538362.2022.2144983

[B16] Lin J. Yu T. Ching L. (2022b). Vitamin C attenuates predisposition to high-fat diet-induced metabolic dysregulation in GLUT10-deficient mouse model. Genes and Nutr. 17, 10. 10.1186/s12263-022-00713-y PMC9288715 35842612

[B17] Lin S. Yang C. Jiang R. Wu C. Lang D. Wang Y. (2024). Flavonoid extracts of Citrus aurantium L. var. amara Engl. Promote browning of white adipose tissue in high-fat diet-induced mice. J. Ethnopharmacol. 324, 117749. 10.1016/j.jep.2024.117749 38219880

[B18] Li R. Zhu Q. Wang X. Wang H. (2022). Mulberry leaf polyphenols alleviated high-fat diet-induced obesity in mice. Front. Nutr. 9, 979058. 10.3389/fnut.2022.979058 36185673 PMC9521161

[B19] Li X. Liu X. Yang F. Meng T. Li X. Yan Y. (2024a). Mechanism of Dahuang Mudan Decotion in the treatment of colorectal cancer based on network pharmacology and experimental validation. Heliyon 10, e32136. 10.1016/j.heliyon.2024.e32136 38882337 PMC11176830

[B20] Li Y. Chen S. Lyu X. Fang X. Cao X. (2024b). Metabolomic analysis to unravel the composition and dynamic variations of anthocyanins in bayberry-soaked wine during the maceration process. Food Chem. X 21, 101175. 10.1016/j.fochx.2024.101175 38379795 PMC10876708

[B21] Lukas L. M. (2023). The potential of plant extracts containing flavonoids as anti-hyperlipidemia: a literature review. Indonesia J. Biomed. Sci. 17, 224–226. 10.15562/ijbs.v17i2.481

[B22] Miguel G. José P. InêS D. M. Dejan S. Marina S. Soković M. (2021). Ultrasound-Assisted extraction of flavonoids from kiwi peel: process optimization and bioactivity assessment. Appl. Sci. 11, 6416. 10.3390/app11146416

[B23] Min Z. Xinyi Q. Tao Y. Zixuan L. Chengfeng L. Shuna T. (2024). Integration of pharmacodynamics, metabolomics and network pharmacology to elucidate the effect of Prunella vulgaris seed oil in the treatment of hyperlipidemia. Arabian J. Chem. 17, 105486. 10.1016/j.arabjc.2023.105486

[B24] Nicolò M. Romina M. Lara C. Andrea M. Anna P. Francesco B. (2014). A new “functional” pasta containing tartary buckwheat sprouts as an ingredient improves the oxidative status and normalizes some blood pressure parameters in spontaneously hypertensive rats. Food and Funct. 5, 1017–1026. 10.1039/c3fo60683j 24658587

[B25] Pan R. Lijuan Y. Yasong C. Qizong C. Yufei X. Qingfu C. (2022). Analysis of total flavonoids, crude protein and its components in different lines of self-fertile common buckwheat, golden tartary buckwheat and rice tartary buckwheat. Guihaia 42, 874–885. 10.11931/guihaia.gxzw202007017

[B26] Ping L. Jianran H. Hongmei Z. Jing F. Baofeng C. (2022). Multi-omics reveals inhibitory effect of baicalein on non-alcoholic fatty liver disease in mice. Front. Pharmacol. 13, 925349. 10.3389/fphar.2022.925349 35784718 PMC9240231

[B27] Ping X. Juxiong L. Zhe L. Xingchi K. Guiqiu H. Yu C. (2023). Tartary buckwheat flavonoids relieve non-alcoholic fatty liver disease by inhibiting lipid accumulation, inflammation, and regulating intestinal flora. Rev. Bras. Farmacogn. 33, 965–979. 10.1007/s43450-023-00406-6

[B28] Qiang X. Xiao J. Xili T. (2023). Development of apple lycium barbarum wine and analysis of volatile components. Sci. Technol. Food Industry 44, 151–159. 10.13386/j.issn1002-0306.2022070048

[B29] Qinyuan L. Peifang W. Zufang W. (2020). Quality and aroma characteristics of honey peach wines as influenced by different maturity. Int. J. Food Prop. 23, 445–458. 10.1080/10942912.2020.1736094

[B30] Sedmak J. J. Grossberg S. E. (1977). A rapid, sensitive, and versatile assay for protein using Coomassie brilliant blue G250. Anal. Biochem. 79, 544–552. 10.1016/0003-2697(77)90428-6 68686

[B31] Siyu H. Wei D. Yanrong H. Yuanhuai H. Hongying L. Longlong L. (2021). Elucidation of the regulatory network of flavonoid biosynthesis by profiling the metabolome and transcriptome in tartary buckwheat. J. Agric. food Chem. 69, 7218–7229. 10.1021/acs.jafc.1c00190 34151566

[B32] Wang L. Liu L. Wu H. Li C. Zhao H. Wu Q. (2023). Evolutionary and expression analysis of starch synthase genes from Tartary buckwheat revealed the potential function of FtGBSSII‐4 and FtGBSSII‐5 in seed amylose biosynthesis. Crop Sci. 63, 2925–2940. 10.1002/csc2.21059

[B33] Wang L. Zhao J. Mao Y. Liu L. Li C. Wu H. (2024). Tartary buckwheat rutin: accumulation, metabolic pathways, regulation mechanisms, and biofortification strategies. Plant Physiology Biochem. 208, 108503. 10.1016/j.plaphy.2024.108503 38484679

[B34] Wu X. Guo T. Li B. Han S. Hu Z. Luo Y. (2024). Parboiled rice supplementation alleviates high-fat diet-induced hyperlipidemia by regulating genes and gut microbiota in mice. Food Sci. Hum. Wellness 13, 1422–1438. 10.26599/fshw.2022.9250120

[B35] Xiaoli Z. Senjie L. Yiming Z. Huan Z. Beibei Y. Hong W. (2022). A metabolomics study of the intervention effect of Tartary buckwheat on hyperlipidemia mice. J. food Biochem. 46, e14359. 10.1111/jfbc.14359 35933651

[B36] Xiaoni Z. Haidong X. Jie W. Ruyue L. Xiaojing Z. Qian G. (2021). Effect of selenium-enriched kiwifruit on body fat reduction and liver protection in hyperlipidaemic mice. Food and Funct. 12, 2044–2057. 10.1039/d0fo02410d 33532813

[B37] Xin Y. Anran Z. Xuanchen L. Shengling H. Yi Z. Wen A. (2023). Effects of extracted oil of fermented Tartary buckwheat on lipid-lowering, inflammation modulation, and gut microbial regulation in mice. Food and Funct. 14, 10814–10828. 10.1039/d3fo04117d 37982812

[B38] Yanbo Liu Y. W. Haideng L. I. Feifei L. I. Song MENGJIAO Zihong L. I. Zhang TAOTAO (2022). Optimization of fermentation technology for composite fruit and vegetable wine by response surface methodology and analysis of its aroma components. RSC Adv. 12, 35616–35626. 10.1039/d2ra04294k 36545074 PMC9745641

[B39] Ying D. Guifang Y. Runli H. Xiaolin Y. Suyun C. Yanqing W. (2022). Identification of candidate genes for easily-shelled traits in Tartary buckwheat based on BSA-Seq and RNA-Seq methods. Euphytica 218, 91. 10.1007/s10681-022-03023-x

[B40] Yuan L. Duan J. (2017). Optimization of enzyme-assisted extraction technology for tartary buckwheat shell procyanidins with response surface methodology. Agric. Sci. and Technol. 18, 1196–1201. 10.16175/j.cnki.1009-4229.2017.07.011

[B41] Yu M. Man Y. Lei R. Lu X. Wang Y. (2020). Metabolomics study of flavonoids and anthocyanin-related gene analysis in kiwifruit (Actinidia chinensis) and kiwiberry (Actinidia arguta). Plant Mol. Biol. Report. 38, 353–369. 10.1007/s11105-020-01200-7

[B42] Zhang J. M. Liang S. Nie P. Liao Y. A. I Q. A. Yan X. (2022). Efficacy of Kushen decoction on high-fat-diet-induced hyperlipidemia in rats. J. traditional Chin. Med. 42, 364–371. 10.19852/j.cnki.jtcm.20220225.002 PMC9924673 35610005

