# Supplementary material for: Transcriptomic and metabonomic profiling unveils the mechanism of Tartary buckwheat and kiwi co-fermentation products in hyperlipidemia treatment
Source: Front Pharmacol. 2025 May 30;16:1572593. doi: 10.3389/fphar.2025.1572593 (PMC12162925; doi:10.3389/fphar.2025.1572593)
Supplement: Supplementary file 5 [file Supplementaryfile1.doc]

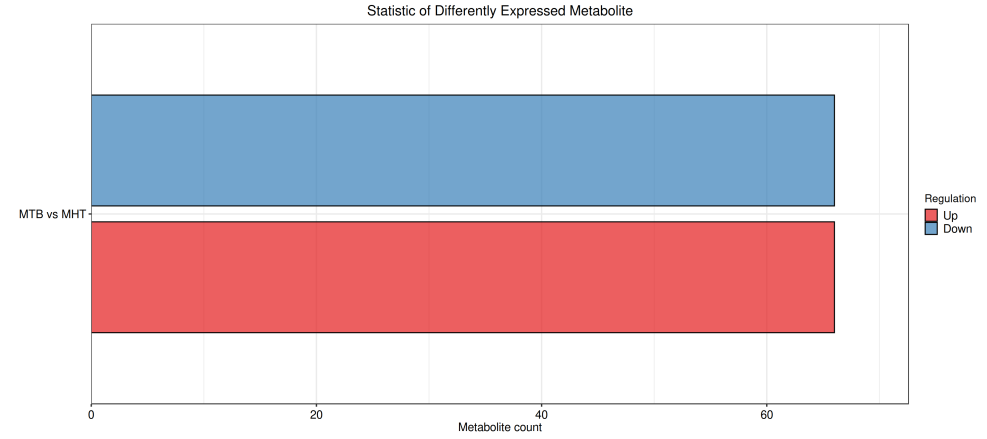


Supplementary Figure 1. Statistic of Differently Expressed Metabolite


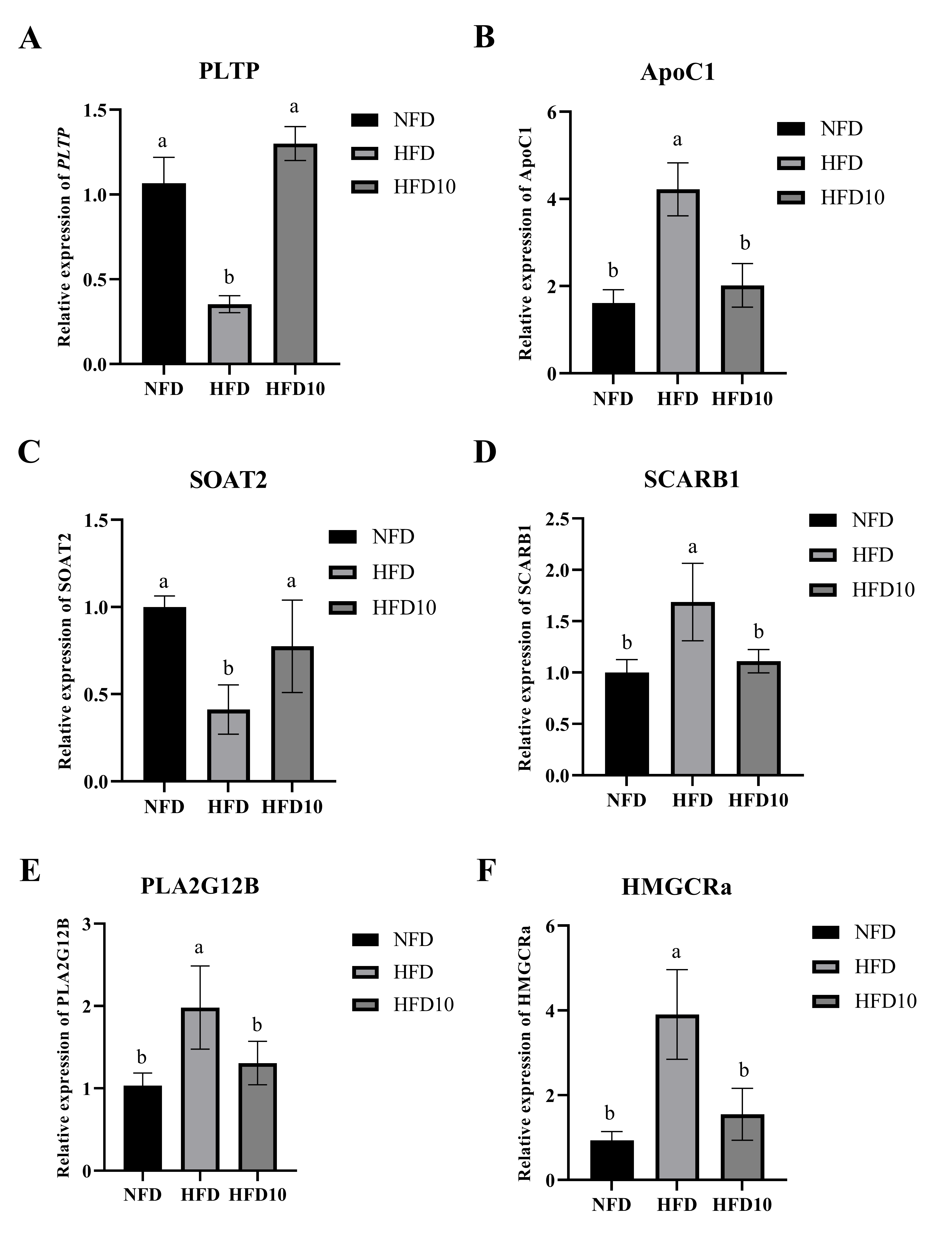


Supplementary Figure 2. Statistic of Differently Expressed Metabolite. Different letters on the data indicate significant differences compared with the other groups (p < 0.05).


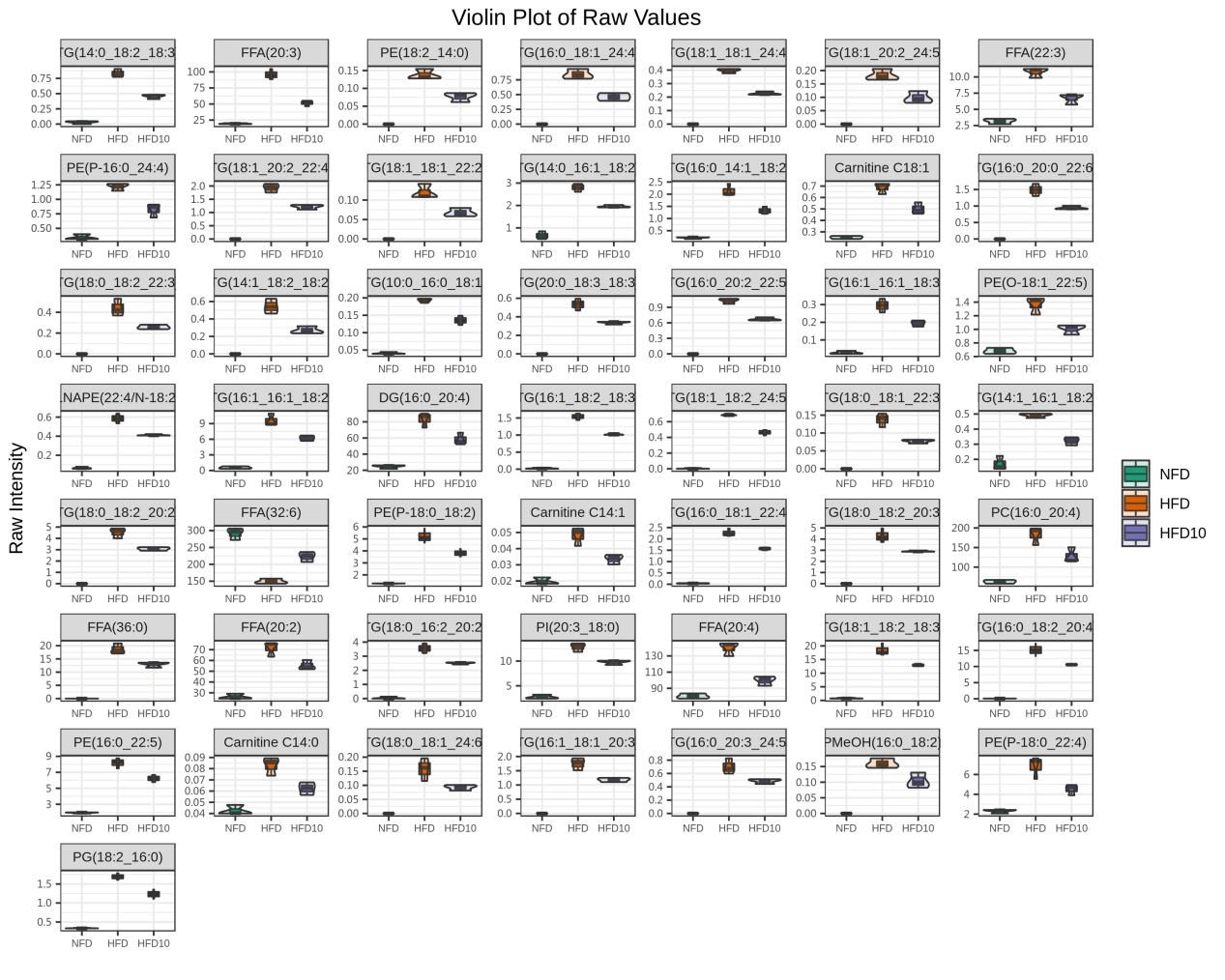


Supplementary Figure 3. Violin Plot of Raw Values

Table S1. Primer sequences

| Primer codes | Primer sequence (5′ – 3′) | Description |
| --- | --- | --- |
| PLTP-F1 | CGTGAACATACCCGTGGATAAG | qPCR primer for PLTP^[1]^ |
| PLTP-R1 | CCTCTAATCGGCAGCTCTGATC |  |
| ApoC1-F | TGCTGTACACACAGCAGAGG | qPCR primer for ApoC1^[2]^ |
| ApoC1-R | TGGAAGGCGGTTTTGGTCTT |  |
| SOAT2-F | TTCTTGTTCTGCCCGACTCT | qPCR primer for SOAT2^[3]^ |
| SOAT2-R | CAAAGCCTGATCCGTCCTG |  |
| SCARB1-F | AATGGGGCTTTTTCACTGGTTGTGC | qPCR primer for SCARB1^[4]^ |
| SCARB1-R | ACATGCAGTACATCTTCATCGCTCTCG |  |
| PLA2G12B-F | CTGCTTGGCTTCCAGTTTGAT | qPCR primer for PLA2G12B^[5]^ |
| PLA2G12B-R | TCTGCAAAGGTCTCACATGC |  |
| HMGCRa-F | CCAGTCAGGAGTGTCCAGGT | qPCR primer for HMGCRa^[6]^ |
| HMGCRa-R | TGCCTGCTTAGTGCATGTTC |  |
| β-actin-F | AGCACGGTATTGTGACTAACTG | qPCR primer for β-actin^[7]^ |
| β-actin-R | TCGAACATGATCTGTGTCATC |  |

Table S2. Comparison table of target sites and compounds

| Corresponding compounds | Class | Target genes |
| --- | --- | --- |
| all-trans-Retinoic acid | Prenol lipids | HMGCR |
| Farnesoic acid |  | HMGCR |
| 3,4-Methylenedioxyamphetamine | Benzodioxoles | HMGCR |
| (13E)-11a-Hydroxy-9,15-dioxoprost-13-enoic acid | Fatty Acyls | HMGCR |
| 1-(3,4-Dihydroxyphenyl)-5-hydroxy-3-decanone | ------ | HMGCR |
| Palmitic acid | Fatty Acyls | HMGCR |
| Cortexolone | Steroids and steroid derivatives | HMGCR |
| Quinoline | Quinolines and derivatives | PLA2G12B |
| Bovinic acid | Fatty Acyls | PLA2G1B |
| Vaccenic acid | Fatty Acyls | PLA2G1B |
| Palmitoleic acid | Fatty Acyls | PLA2G1B |
| 3,4-Dihydroxybenzeneacetic acid | Phenols | PLA2G1B |
| 2-Methylbenzoic acid | Benzene and substituted derivatives | PLA2G1B |
| Phenylpyruvic acid | Benzene and substituted derivatives | PLA2G1B |
| 1-Hexadecanol | Fatty Acyls | PLA2G1B |
| Kaempferide | Flavonoids | PLA2G1B |
| Quercetin | Flavonoids | PLA2G1B |
| Luteolin | Flavonoids | PLA2G1B |
| Naringenin | Flavonoids | PLA2G1B |
| 4-Hydroxycinnamoylagmatine | Cinnamic acids and derivatives | PLA2G1B |
| 11-Dehydrocorticosterone | Steroids and steroid derivatives | PLA2G1B |
| 12-Keto-tetrahydro-leukotriene B4 | Fatty Acyls | PLA2G4B |
| Indole | Indoles and derivatives | SCARB1 |
| Mirtazapine | Piperazinoazepines | SOAT1 |
| 2-Phenylethanol | Benzene and substituted derivatives | SOAT1 |
| Alpha-Tocotrienol | Prenol lipids | SOAT1 |
| (6Z)-Octadecenoic acid | ------ | SOAT1 |
| Phytosphingosine | Organonitrogen compounds | SOAT1 |
| Sphinganine | Organonitrogen compounds | SOAT1 |
| Octadecanamide | Fatty Acyls | SOAT1 |

**REFERENCE**

[1] Shogo M, Nabila T, S P I. Bpifcl Modulates Kiss2 Expression under the Influence of 11-Ketotestosterone in Female Zebrafish [J]. Scientific reports, 2017, 7(1-4): 7926.

[2] Whitney T, J E E, P F A*, et al.* Modulation of Retinoid-X-Receptors Differentially Regulates Expression of Apolipoprotein Genes Apoc1 and Apoeb by Zebrafish Microglia [J]. Biology open, 2022, 11(1): bio058990.

[3] Chang N, Chan Y, Ding S*, et al.* Sterol O-Acyltransferase 2 Contributes to the Yolk Cholesterol Trafficking During Zebrafish Embryogenesis [J]. PLoS ONE, 2016, 11(12): 1-24.

[4] F V R A, Lindsay M, J R F*, et al.* Stabilin 1 and 2 Are Important Regulators for Cellular Uptake of Apolipoprotein B-Containing Lipoproteins in Zebrafish [J]. Atherosclerosis, 2022, 346: 18-25.

[5] Junli F, Xi C, Shitong W*, et al.* Transcriptomics Integrated with Metabolomics Reveals the Ameliorating Effect of Mussel-Derived Plasmalogens on High-Fat Diet-Induced Hyperlipidemia in Zebrafish [J]. Food & function, 2023, 14(8): 3641-3658.

[6] Zhiqi L, Yang T, Zhenquan L*, et al.* Hepatotoxicity Induced by Ppⅵ and Ppⅶ in Zebrafish Were Related to the Cholesterol Disorder [J]. Phytomedicine : international journal of phytotherapy and phytopharmacology, 2021, 95: 153787-153787.

[7] Xue H, Xiaowen X, Tingting Y*, et al.* Diflubenzuron Induces Cardiotoxicity in Zebrafish Embryos [J]. International Journal of Molecular Sciences, 2022, 23(19): 11932-11932.
